# Functional diversification of duplicate genes through subcellular adaptation of encoded proteins

**DOI:** 10.1186/gb-2008-9-3-r54

**Published:** 2008-03-12

**Authors:** Ana C Marques, Nicolas Vinckenbosch, David Brawand, Henrik Kaessmann

**Affiliations:** 1 Center for Integrative Genomics, University of Lausanne, CH-1015 Lausanne, Switzerland

## Abstract

**Background:**

Gene duplication is the primary source of new genes with novel or altered functions. It is known that duplicates may obtain these new functional roles by evolving divergent expression patterns and/or protein functions after the duplication event. Here, using yeast (*Saccharomyces cerevisiae*) as a model organism, we investigate a previously little considered mode for the functional diversification of duplicate genes: subcellular adaptation of encoded proteins.

**Results:**

We show that for 24-37% of duplicate gene pairs derived from the *S. cerevisiae *whole-genome duplication event, the two members of the pair encode proteins that localize to distinct subcellular compartments. The propensity of yeast duplicate genes to evolve new localization patterns depends to a large extent on the biological function of their progenitor genes. Proteins involved in processes with a wider subcellular distribution (for example, catabolism) frequently evolved new protein localization patterns after duplication, whereas duplicate proteins limited to a smaller number of organelles (for example, highly expressed biosynthesis/housekeeping proteins with a slow rate of evolution) rarely relocate within the cell. Paralogous proteins evolved divergent localization patterns by partitioning of ancestral localizations ('sublocalization'), but probably more frequently by relocalization to new compartments ('neolocalization'). We show that such subcellular reprogramming may occur through selectively driven substitutions in protein targeting sequences. Notably, our data also reveal that relocated proteins functionally adapted to their new subcellular environments and evolved new functional roles through changes of their physico-chemical properties, expression levels, and interaction partners.

**Conclusion:**

We conclude that protein subcellular adaptation represents a common mechanism for the functional diversification of duplicate genes.

## Abstract 2

Analysis of the subcellular localization patterns of duplicate genes revealed that protein subcellular adaptation represents a common mechanism for the functional diversification of duplicate genes.

## Background

Gene duplication is an important evolutionary mechanism, providing genomes with the genetic raw material for the emergence of genes with new or altered functions [1]. Several evolutionary fates of the two duplicate gene copies are possible and have been described. For instance, one of the two copies may be redundant and accumulate deleterious mutations that eventually render it a non-functional pseudogene [1]. Alternatively, both copies might be functionally preserved by natural selection if an increase in gene dosage of the ancestral gene is beneficial [2], or if a change of the stoichiometry of proteins in complexes (for example, after whole genome duplication (WGD) events) would be deleterious [3,4]. Finally, if both gene copies are preserved after the duplication event, they may functionally diverge in two major ways.

In the classic scenario termed neofunctionalization [1], one of the duplicates evolves a new function (usually defined as a new biochemical function of the encoded protein), while the other retains the ancestral function of the progenitor gene. An alternative model - termed subfunctionalization - posits that the ancestral functions are partitioned between the two duplicates such that their joint levels and patterns of activity are equivalent to the single ancestral gene [5-7]. 'Gene function' in this model is defined as either a function of the encoded proteins [6,8] or the expression pattern of the gene [5,9]. In addition, a combination of these two scenarios ('subneofunctionalization') was recently proposed [10].

The subcellular localization of a protein is key to its function in the cell [11]. In view of this and prompted by the observation that a number of individual reports describe gene families that encode proteins that differ with respect to their subcellular localization (see, for example, [12,13]; for more individual examples, see also [14]), we set out to systematically investigate an - as yet - little considered alternative mechanism for the functional diversification of duplicate genes, namely, the subcellular relocalization and adaptation of their encoded proteins [14] (which may or not be followed or accompanied by changes of gene expression patterns and/or functional/biochemical properties of the proteins).

To this end, we used the yeast *Saccharomyces cerevisiae *as a model, for three reasons. First, the subcellular localization of a large proportion (approximately 75%) of its proteins was recently established [15]. Second, in addition to other duplicates, the WGD event in this species, which occurred approximately 100 million years ago [16], resulted in a large set of well-defined duplicate gene pairs with the same age (that is, they have the same divergence time). Finally, a wide range of genome- and proteome-wide functional data sets are available for this organism. Thus, the *S. cerevisiae *genome/proteome provides a unique opportunity to assess the extent and patterns of protein subcellular adaptation after gene duplication.

## Results and discussion

### Subcellular divergence is common among yeast whole-genome duplicates

Using protein localization data (22 compartments; obtained by green fluorescent protein (GFP)-fusion analysis) covering 75% of the *S. cerevisiae *proteome [15], we established the subcellular localization of proteins encoded by 900 yeast genes, forming 450 pairs of WGD-derived duplicate genes [17] (see Materials and methods for details). Among these, we collected 238 pairs for which both paralogs are unambiguously assigned to at least one subcellular compartment (Table 1; see Materials and methods). For 88 of these protein pairs (approximately 37%), we found that the two duplicates are located in at least one different subcellular compartment (Table 1 and Additional data file 1).

**Table 1:** Subcellular localization data for *S. cerevisiae *proteins in this study

	Number of *S. cerevisiae *proteins*	Number of WGD duplicate pairs^†^	Number of WGD pairs with distinct localizations for the two members
GFP tagging^‡^	3,919 (62.9%)	238 (52.8%)	88 (37.0%)
Epitope tagging^§^	2,745 (44.0%)	124 (27.5%)	53 (42.7%)
GFP/epitope tagging overlap^¶^	2,716 (43.5%)	75 (16.7%)	18 (24%)

*Out of 6,234 annotated yeast open reading frames [15]. ^†^Out of 450 gene pairs [17]. ^‡^Based on [15] unambiguous protein localization assigments. ^§^Based on [19]. ^¶^Gene pairs for which the subcellular localization is unambiguously assigned to both paralogs in both datasets.

The localization data we used was previously shown to be in 80% agreement with data (small and large-scale) from the *Saccharomyces *Genome Database [15,18], suggesting that the subcellular assignments are generally reliable. However, to assess to what extent experimental artifacts may potentially have influenced the analysis of subcellular divergence between duplicates, we performed a second analysis using earlier *S. cerevisiae *localization data generated by epitope-tagging [19]. The two localization analyses present considerable differences in their experimental setup and the number of cellular compartments covered. Thus, the error sources and potentially misassigned subcellular localizations are expected to be different between the two datasets (for details see [15]).

We found no significant difference in the proportion of paralogous protein pairs showing distinct subcellular localizations between the GFP (88 of 238 pairs, approximately 37%) and epitope data (53 of 124 pairs, approximately 43%; two-tailed *P *= 0.31, Fisher's exact test; Table 1). We also considered 75 paralogous protein pairs for which localization was assigned in both the GFP and the epitope fusion analyses. Among these, 18 (24%) showed a distinct subcellular localization in both experimental sets (Table 1). Thus, we estimate that approximately 24-37% of the *S. cerevisiae *WGD pairs show protein localization differences, consistent with a recent estimate (approximately 19%) based on Gene Ontology (GO) annotation [20]. This suggests that a significant proportion of yeast duplicates have diverged in terms of their subcellular localization.

All following analyses are based on the GFP-fusion localization data [15], since they represent the most extensive and reliable localization survey of the budding yeast proteome available. WGD-derived duplicates with distinct cellular localization will be referred to as D-pairs, and those with the same subcellular distribution as S-pairs.

### Subcellular localization change and protein function

As some biological processes (for example, phosphorylation) are widespread in the cell, whereas others, such as transcription, are restricted to certain organelles (nucleus, mitochondria), one may expect that ancestral functions may impose different constraints with respect to the subcellular diversification potential of duplicates.

To assess whether a gene's biological function indeed influences the subcellular localization fate of proteins after duplication, we tested for general functional differences between genes in S- and D-pairs using GO annotation. In this analysis, we assume that the current GO distribution of duplicates overall reflects that of their ancestors. Two GO categories stand out (Table 2). While S-pairs show a significant excess of genes involved in biosynthetic processes, D-pairs are significantly enriched with genes involved in catabolism (*P *≤ 0.01 after false discovery rate correction [21]). We note that, generally, *S. cerevisiae *proteins (excluding the WGD duplicates) involved in catabolism are located in, on average, 1.47 compartments, while those that contribute to biosynthesis localize in 1.35 compartments, a significantly different distribution (two-tailed *P *< 0.01, Mann-Whitney *U *test). This suggests that the *a priori *wider subcellular distribution of proteins involved in catabolic pathways facilitates functional divergence through subcelullar relocalization after gene duplication when compared to biosynthetic proteins, which show more restricted localization patterns.

**Table 2:** Summary of GO analysis for D- and S-pair duplicates

	D-pair		S-pair			
				
Biological process*	No.	Percentage	No.	Percentage	*P*-value^†^	Excess^‡^
Biosynthetic process	32	21.1	111	42.9	0.0008	56
Catabolic process	32	21.1	17	6.6	0.0024	22
Regulation of biological process	59	38.8	56	21.6	0.0172	26
Response to biotic stimulus	0	0.0	12	4.6	0.1480	12
Nitrogen compound metabolic process	4	2.6	21	8.1	0.3988	14
Cell communication	18	11.8	15	5.8	0.4587	9
Primary metabolic process	110	72.4	210	81.1	0.5012	23
Cell cycle	21	13.8	20	7.7	0.5012	9
Response to endogenous stimulus	12	7.9	9	3.5	0.5012	7
Chemical homeostasis	0	0.0	6	2.3	0.5012	6
Cellular developmental process	11	7.2	9	3.5	0.5012	6
Chromosome segregation	5	3.3	2	0.8	0.5012	4
Cell division	21	13.8	25	9.7	0.6028	6
Response to stress	24	15.8	29	11.2	0.6483	7
Reproductive process	14	9.2	15	5.8	0.6696	5
Conjugation	7	4.6	6	2.3	0.6982	3
Sexual reproduction	7	4.6	6	2.3	0.6982	3
Aging	1	0.7	6	2.3	0.7084	4
Cellular metabolic process	120	79.0	214	82.6	0.7686	9
Asexual reproduction	3	2.0	2	0.8	0.7686	2
Nuclear division	1	0.7	0	0.0	0.7686	1
Protein localization	10	6.6	23	8.9	0.7785	6
Cell adhesion	0	0.0	2	0.8	0.7785	2
Response to chemical stimulus	17	11.2	25	9.7	0.8583	2
RNA localization	6	4.0	9	3.5	0.9959	1
Anatomical structure development	12	7.9	19	7.3	1	1
Establishment of localization	33	21.7	55	21.2	1	1
Macromolecule metabolic process	98	64.5	169	65.3	1	2
Response to external stimulus	0	0.0	1	0.4	1	1
Maintenance of localization	1	0.7	1	0.4	1	0
Response to abiotic stimulus	4	2.6	7	2.7	1	0
Cell organization and biogenesis	57	37.5	97	37.5	1	0
Regulation of biological quality	8	5.3	14	5.4	1	0
Autophagy	0	0.0	1	0.4	1	1
Filamentous growth	8	5.3	13	5.0	1	0
Cell homeostasis	7	4.6	12	4.6	1	0
Regulation of a molecular function	1	0.7	3	1.2	1	1
Cell proliferation	0	0.0	1	0.4	1	1
Non-developmental growth	1	0.7	2	0.8	1	0

*We selected GO level 3, since this constitutes a good compromise between the number of genes annotated and the depth of the information contained in each class [46]. ^†^After false discovery rate correction. ^‡^Represents the difference between the observed number of genes in the over-represented set and what would be expecated based on the observed genes in the other gene set.

**Figure 1:**
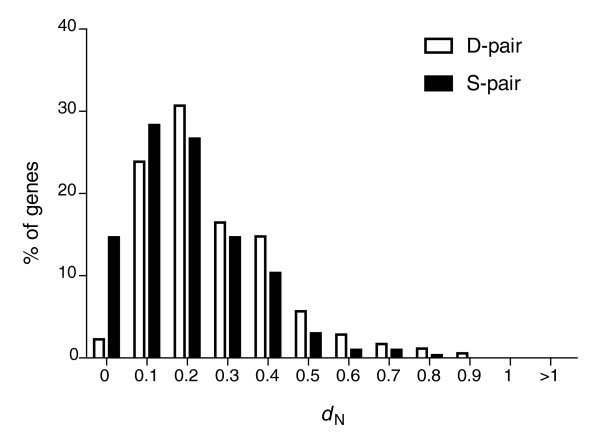
Distribution of non-synonymous substitution rates (*d*_N_) for duplicate genes in S- and D-pairs (estimated for the time since the whole-genome duplication event - see text for details).

Next, we analyzed the extent of amino acid divergence in D- and S-pair duplicates. To this end, we used a related yeast species, *Kluyveromyces waltii *[17], which diverged from *S. cerevisiae *before the WGD event, as an outgroup, and estimated the non-synonymous substitution rate (that is, the number of non-synonymous substitutions per non-synonymous site, *d*_N_) on the lineages leading to each one of the two *S. cerevisiae *duplicates using a maximum-likelihood approach [22] (see Materials and Methods for details). This analysis revealed a difference in the *d*_N _distribution between genes in S- and D-pairs (Figure 1; Additional data file 1; two-tailed *P *< 10^-5^, Mann-Whitney *U *test); S-pair genes generally show lower non-synonymous substitution rates than those in D-pairs.

Consistent with previous observations [17], cases of extreme decelerated evolution (one of the duplicates has a *d*_N _= 0) among S-pairs include protein coding genes that are known to be highly constrained, such as ribosomal genes (28 pairs), histones (2 pairs) and elongation factors (2 pairs). Selection for increased gene dosage and/or decreased dosage imbalance may explain the intensity of purifying selection observed for these 'housekeeping' duplicates [1,3,23]. The fact that these duplicates did not change their subcellular localization is likely due to the specificity of their biological function, which is restricted to certain compartments and may generally preclude subcellular shifts, as suggested by our data above.

We also found that S-pair genes show higher expression levels than D-pair genes (median = 1.3 copies per cell versus 0.8 copies per cell; two-tailed *P *< 10^-5^, Mann-Whitney *U *test), consistent with the idea that many S-pairs represent duplications of housekeeping genes. This difference is also reflected at the protein level; D-pair genes (median = 5,436.9 pmol) express significantly more protein than S-pair genes (mean = 35,788.3 pmol, two-tailed *P *< 0.01, Mann-Whitney *U *test).

Thus, generally, biological function appears to be a strong determinant for the propensity of duplicates to relocate in the cell. While duplicate proteins encoded by slowly evolving housekeeping genes with high expression levels (for example, genes involved in biosynthetic process, such as ribosomal genes) tend to preserve ancestral localization patterns (and functions) after duplication, duplicates from other categories, such as those involved in catabolic processes, are much more likely to evolve divergent localization patterns.

### Functional divergence of duplicates through neo- or sublocalization

**Figure 2:**
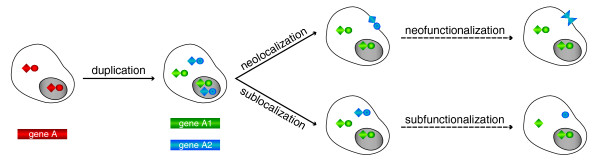
Illustration of the different evolutionary fates of (functional) duplicate genes. Each gene/protein is represented in different colors: red, ancestral, 'A'; green, duplicate copy A1; and blue, duplicate copy A2. Different shapes of proteins (circle, square, and triangle) indicate different functions. Three different subcellular localizations (nucleus, cytoplasm, and cytoplasmic membrane) are indicated in a schematic cell. We note that only the major possible scenarios are illustrated here.

But how do these divergent localization patterns emerge? Akin to concepts proposed for the functional divergence of duplicate genes through changes in expression and/or protein function, we hypothesized that duplicates may show two types of subcellular divergence (Figure 2). First, ancestral cellular compartments may be partitioned between them, or they may specifically localize to only part of the ancestral compartments, a process we term 'sublocalization'. Second, they may localize to new, previously unoccupied compartments ('neolocalization'). These two processes are analogous to the traditional neo/subfunctionalization concepts [1,5-7,9], but should be treated separately, as neo/subfunctionalization of the biochemical function and/or expression of the duplicate (that is, the previously considered fates of duplicates) may follow or accompany subcellular divergence (Figure 2).

If divergent subcellular localization between duplicates was a consequence of sublocalization alone, the joint number of different compartments per protein pair (that is, combining both duplicates) would be expected to be the same as that of the common ancestral protein. Conversely, the number of compartments per pair should be higher than that of the progenitor if neolocalization contributed to subcellular diversification.

To assess the contribution of neo- and sublocalization to the functional diversification of duplicates and given the lack of subcellular localization data for ancestral proteins, we used the average number of subcellular compartments of yeast singleton gene products (that is, genes that show no evidence of paralogs in the *S. cerevisiae *genome; see Materials and methods for details) as a proxy for the subcellular representation of WGD duplicate progenitors (akin to a previous analysis of yeast duplicates [10]).

We observed that the joint number of distinct compartments per D-pair (mean = 2.31 ± 0.63, median = 2) is significantly higher than that observed for singleton proteins (mean = 1.30 ± 0.49, median = 1, two-tailed *P *< 10^-5^, Mann-Whitney *U *test). In contrast, there is no difference between the distributions of the number of subcellular compartments for S-pairs (mean = 1.27 ± 0.42, median = 1) and singletons (two-tailed *P *= 0.2, Mann-Whitney *U *test), suggesting that the increase in the number of compartments observed for D-pairs is due to neolocalization events among D-pair proteins.

A potential caveat of this analysis is that the types of proteins represented in D-pairs might generally and *a priori *be present in a larger number of compartments, as also indicated by the analysis of the number of compartments for catabolic/biosynthetic proteins discussed above. To control for this, we compared the number of distinct compartments per D-pairs and singletons for proteins within the same GO classes. To ensure adequate sample sizes, we focused on the 8 GO categories that contain more than 30 proteins for both D-pairs and singletons (Table 3). This analysis shows that for all eight comparisons, the joint number of compartments per D-pair is significantly higher than that observed for singletons (Table 3; two-tailed *P *< 10^-4^, Mann-Whitney *U *test). This suggests that the elevated number of compartments for D-pairs is indeed a result of neolocalization and not due to a wide cellular representation of ancestral progenitor proteins, prior to duplication. Based on the observed excess (mean, approximately 0.98) of D-pairs relative to singletons from the same functional categories, we estimate that, on average, approximately one compartment is gained by neolocalization per duplication event between duplicates showing subcellular divergence. In addition to the elevated number of compartments per D-pairs, we find that the average number of compartments per D-pair protein (approximately 1.53) is significantly higher than that of singletons in 7 of 8 comparisons (two-tailed *P *< 0.05, Mann-Whitney *U *test). This result further underscores that neolocalization probably predominated over sublocalization during yeast duplicate evolution.

**Table 3:** Comparison between the number of different compartments per D-pairs, proteins in D-pairs, and singleton proteins from the same GO categories

	Singleton		D-protein			D-pair		
			
Biological process*	Total no.	Average no. of compartments	Total no.	Average no. of compartments	*P*-value^†^	Total no.	Average no. of compartments	*P*-value^‡^
Regulation of biological process	64	1.30	59	1.56	0.020	32	2.31	1.92E-10
Macromolecule metabolic process	247	1.28	98	1.54	0.001	52	2.31	1.57E-19
Cell organization and biogenesis	160	1.28	57	1.60	0.021	37	2.38	2.27E-14
Primary metabolic process	325	1.30	110	1.52	0.005	57	2.28	1.93E-20
Catabolic process	53	1.47	32	1.41	0.370	16	2.19	1.19E-04
Establishment of localization	81	1.17	33	1.55	0.013	19	2.37	1.82E-09
Biosynthetic process	162	1.30	32	1.53	0.044	18	2.11	1.52E-07
Cellular metabolic process	372	1.32	120	1.52	0.006	62	2.27	1.42E-21
Regulation of biological process	64	1.30	59	1.56	0.020	32	2.31	1.92E-10

The data are derived from 8 categories with at least 30 proteins per group - see text for details. *GO level 3. ^†^Mann-Whitney *U *test comparing D-proteins and singletons. ^‡^Mann-Whitney *U *test comparing D-pairs and singletons.

**Figure 3:**
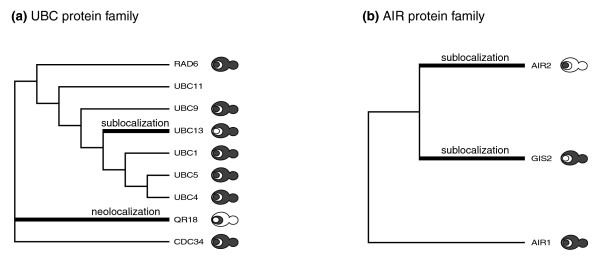
Subcellular localizations of the **(a) **UBC and **(b) **AIR family members and subcellular localization changes inferred based on the phylogeny. The common name and yeast protein identifier (in brackets) of the protein are indicated. The schematic representation of a yeast cell depicts three possible localizations: nucleus (small circle), endoplasmatic reticulum (eclipse around nucleus), and cytoplasm (remainder of the cell). The co-localization of the protein with one of the yeast subcellular compartments is indicated by grey shading.

To further assess and illustrate the types and extent of subcellular relocalizations in the evolution of yeast gene families, we used the *K. waltii *ortholog(s) as outgroups and reconstructed the phylogeny for 45 WGD yeast families (15 D- and 30 S-pair-containing gene families) with at least 1 additional member and mapped the subcellular localizations of these onto the phylogenies (Figure 3 and Additional data file 2). In 16 families, the subcellular localization has remained completely preserved among the members (Additional data file 2). For the remaining 29 families, we analyzed changes in protein location, assuming that the scenario requiring the smallest number of subcellular changes, given the observed data (parsimony principle), reflects the true pattern of events.

For 16 of the 29 families, we could infer the most likely scenario of subcellular diversification. Eight families show instances of neolocalization (Additional data file 2). For example, members of the ubiquitin-conjugating enzyme family, involved in protein degradation [24], are generally located in the cytoplasm and the nucleus (Figure 3a). However, UBC7p (also known as QR18) neolocalized to the endoplasmic reticulum (ER; Figure 3a), where it became essential for the degradation of misfolded proteins [25].

Sublocalization events occurred in four protein families. For instance, GIS2 (cytoplasmic) and AIR2 (nuclear) of the AIR protein family partitioned their ancestral compartments (the cytoplasm and nucleus - still seen for their AIR1 paralog; Figure 3b). Consistent with their specific localizations, GIS2 specialized in a function in the RAS/cAMP signaling pathway [26], whereas AIR2 became specifically involved in the processing and export of mRNAs from the nucleus [27].

In the remaining four families, both neo- and sublocalization events appear to have occurred (Additional data file 2). In addition to the neolocalization event described above, the UBC family also reveals an instance of sublocalization based on GFP data; UBC13 lost the ancestral nuclear localization (Figure 3a). Thus, the UBC family shows both neo- and sublocalization of family members.

### Subcellular shifts and signal peptide evolution

The information required for sorting of proteins to different cellular compartments is encoded in their sequence, sometimes in distinct targeting motifs [11]. Consequently, differences in the subcellular localization of paralogous proteins should be due to protein sequence changes and should, in principle, be detectable. However, the identification of protein targeting sequence determinants has proven to be a difficult task [11]. To elucidate the molecular basis of subcellular relocalization of duplicates, we focused our analysis on the best characterized targeting sequences; amino-terminal signal peptides (SPs) that target proteins to the mitochondria or the ER. These types of SPs are typically 13-36 amino acids long and are usually cleaved from the mature peptide [28].

We estimated the amino acid divergence between WGD proteins pairs with ER and/or mitochondrial localization (36 pairs in total, among which 21 are S-pairs and 15 are D-pairs; Table 4). We then determined the aminoacid divergence in the first either 13 or 36 amino acids (putative signal peptide region) and in the mature peptide (protein sequence without signal peptide), and then compared it between the 21 S- and 15 D-pairs (Table 4).

**Table 4:** Amino acid divergence between WGD protein pairs

	D-pairs	S-pairs	*P*-value*
**Amino-teminus signal peptide**			
13 amino acids	0.92	0.69	0.019^†^
36 amino acids	0.86	0.67	0.017^†^
			
**Mature peptide**			
13 amino acids	0.57	0.50	0.596
36 amino acids	0.57	0.48	0.785

*Two-tailed Mann-Whitney *U *test. ^†^Significant at the 5% level.

The average amino acid divergence in the SP is higher for protein pairs for which the ER/mitochondrial localization is not preserved (the median divergence based on the 13 amino acid SP is 0.92, and based on the 36 amino acid SP is 0.86) than for those pairs that maintained the same subcellular localization (13 amino acid SP, 0.69; 36 amino acid SP, 0.67), a significantly different distribution (two-tailed *P *< 0.05, Mann-Whitney *U *test). In contrast, we observed no significant difference for the accumulation of amino acid substitutions in the mature peptide (for neither of the mature peptide sizes tested) between the two sets of proteins (two-tailed *P *> 0.6, Mann-Whitney *U *test; Table 4), which excludes the possibility that proteins that changed their subcellular localization generally show a faster rate of protein evolution and, therefore, show an elevated SP divergence. Thus, at least for proteins targeted to the mitochondria and to the ER, differences in subcellular localization between duplicates are associated with accelerated signal peptide sequence evolution. Conceivably, this acceleration may have been driven by positive Darwinian selection. A recent study demonstrating selectively driven optimization of a mitochondrial targeting signal of a protein from a young primate gene (L Rosso and colleagues, unpublished) suggests that this is a plausible scenario.

**Figure 4:**
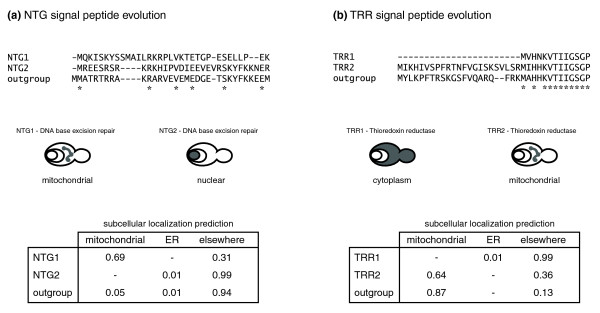
Subcellular relocalization and signal peptide evolution. Signal peptides (36 amino-terminal residues) and experimentally determined subcellular localizations of the **(a) **NTG1/NTG2 and **(b) **TRR1/TRR2 duplicate pairs (derived from the *S. cerevisiae *WGD event) are shown. *K. waltii *orthologous sequences are used as outgroups. Predotar [39,40] was used to predict subcellular localizations based on the protein sequences. The (predicted) subcellular localization of the *K. waltii *proteins was considered to represent the ancestral state. Identical residues in all peptide sequences are represented with (*) under the corresponding position in the protein alignment.

The NTG1/2 base excision repair and TRR1/2 thioredoxin reductase WGD gene pairs provide striking examples of how subcellular reprogramming through changes in targeting sequences may occur (Figure 4). Through a comparison with the NTG orthologous protein from *K. waltii*, we determined that NTG1 gained an amino-terminal signal after the WGD event (mainly through a number of amino acid substitutions) that targets it to mitochondria, while NTG2 maintained the ancestral nuclear localization (Figure 4a). Conversely, TRR1 lost the ancestral capacity to localize to mitochondria, due to a deletion of its amino-terminal mitochondrial targeting sequence (Figure 4b). Thus, while keeping their ancestral enzymatic functions [29,30], both NTG1 and TRR1 obtained new functional roles through neolocalization changes, caused by gain and loss of (mitochondrial) targeting sequences, respectively.

### Functional adaptation to new subcellular environments

Functional adaptation of duplicate proteins to new subcellular compartments may occur in several ways. Given that organelles generally display distinct physico-chemical properties that are reflected in the properties of their proteome and transcriptome [31,32], relocalized duplicates may show physico-chemical adaptations that allow them to optimally function in their new (in the case of neolocalization) or more restricted (sublocalization) cellular environments.

We first tested whether duplicate proteins reveal evidence for adaptation to the pH of the compartments to which they localize. To this end, we analyzed the pI (isoelectric point) of duplicates, since the pI distribution of proteins is specific to compartments and likely associated with the compartments' pH [32]. We observed a significantly different distribution of fold differences in pI between D- and S-pair duplicates (two-tailed *P *< 10^-3^, Mann-Whitney *U *test; see Additional data file 1 for individual values), with D-pairs displaying a higher median fold difference between the members of the pair (0.09) than S-pair genes (0.04). This result is in accordance with the notion that duplicates with different subcellular localization show pI adaptation, likely due to the pH of their new/altered cellular environments. However, alternatively, it remains possible that the elevated pI divergence of D-pair proteins may simply reflect the generally higher amino acid divergence observed for D-pair relative to S-pair duplicates (see above).

To distinguish between these two possibilities, we tested whether the observed substitutions between proteins are biased in terms of the pI of the accumulated amino acids. This analysis revealed that 24 of the 88 D-pairs display a significantly skewed accumulation of substitutions regarding the pI of their amino acids (*P *< 0.05, Pearson's chi-square test; Bonferroni-corrected for multiple (238) tests). In other words, for 24 pairs, the two paralogs have accumulated a significantly larger number of amino acids with a higher or smaller pI, respectively, than expected by chance for such a pairwise comparison (50%). This is a significantly higher proportion of pairs (one-tailed *P *< 0.05, Fisher's exact test) compared to that of S-pairs (26/150 pairs), for which the difference in pI cannot be explained by subcellular localization differences. These analyses suggest that D-pair proteins show adaptation to the pH/pI properties of new or altered cellular environments through the fixation of certain amino acids by natural selection.

The expression level of a gene was reported to also be related - at least in part - to the subcellular localization of its product [31]. This may be due to the different volumes of the various compartments (for example, larger compartments would require more protein molecules according to this hypothesis [31]). We computed the fold difference in mRNA transcript abundance [33] for our set of yeast duplicates. The average expression difference between genes in D-pairs (mean, 0.88) is higher than that between S-pair genes (0.71), a significantly different distribution (two-tailed *P *< 0.05, Mann-Whitney *U *test). The elevated expression divergence of D-pair duplicates may indicate that they generally adapted to the expression level requirements of their compartments, for example, through changes in their regulatory sequences.

### Subcellular adaptation, protein-protein interactions, and the evolution of new functions

The subcellular localization of a protein determines its ability to interact with other proteins in its local environment. Therefore, subcellular diversification of duplicates should often entail changes in their interactions with other proteins. In the case of sublocalization, the descendant duplicate (assuming that it required protein partners for functioning) is bound to lose interaction partners that were specific to the lost compartment(s). Conversely, proteins that occupy new subcellular niches may obtain new interaction partners.

**Figure 5:**
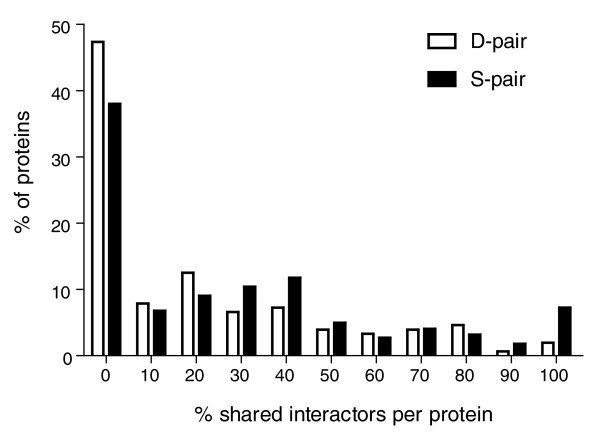
Distribution of the proportion of shared interactors for genes in S- and D-pairs.

Using a database containing extensive *S. cerevisiae *protein interaction data [34], we observed that the two members in D-pairs share a significantly smaller fraction of interactors (median = 6.4%) than duplicates in S-pairs (median = 13.7%, two-tailed *P *< 0.05, Mann-Whitney *U *test; Figure 5). This result is likely not due to a difference in the number of different interactors determined for the two sets of protein pairs (median = 9 and 8 interactors per S- and D-pairs, respectively; two-tailed *P *= 0.48, Mann-Whitney *U *test). Thus, as predicted, subcellular divergence of duplicates appears to lead to a pronounced divergence in terms of their interaction with other proteins.

Subcellular relocalization may allow for the possibility that duplicate proteins evolve new functions (in the case of neolocalization) or functionally specialize (in the case of sublocalization, where both duplicates localize to distinct compartments) by evolving interactions with proteins that are located in their own compartment(s) but not in that of their duplicate copies. To test this, we assessed how often an interactor is located in the same compartment as the D-pair duplicate with which it interacts. We then compared this value to the extent of co-localization of these interactors with the other protein of the pair (with which no interaction was found).

For 1,270 interactions that are not shared between D-pair proteins (involving 955 interactors and 82/88 D-pair proteins), 684 show co-localization of the interactor and the duplicate with which it interacts. This represents a significantly larger overlap than that observed between these interactors and the non-interacting paralogs of the pairs (582/1,270, two-tailed *P *< 10^-4^, Fisher's exact test). We note, however, that - as expected (given the shared history of the two duplicates of a D-pair) - this subcellular overlap is greater than that observed between random protein pairs (1,354/2,628, two-tailed *P *< 10^-4^, Fisher's exact test). These results support the notion that subcellular diversification allowed duplicates to obtain new functions and/or functionally specialize by evolving interactions with proteins that are specific to their compartment(s). Given that neolocalization seems frequent (see above), duplicates appear to often have obtained novel functional roles by evolving interactions with compartment-specific proteins - unattainable to their single copy progenitors.

## Conclusion

In this study, we have begun to assess the role of subcellular relocalization and adaptation for the emergence of new or altered gene functions after duplication, using yeast as a model organism. Our work suggests that subcellular divergence has played a significant role for the functional divergence of duplicate genes. It has affected roughly one-third of yeast WGD duplicates, in particular those involved in biological processes with a wider subcellular distribution (for example, catabolism).

Although subcellular redistribution of duplicate proteins involved repartitioning/loss of ancestral compartments, relocalization of proteins to previously unoccupied compartments (neolocalization) seems to have prevailed and led to an overall gain of compartments among duplicates. Thus, duplicate genes appear to frequently have obtained new functional roles through the process of subcellular relocalization. The finding that relocalized proteins have obtained new interaction partners and lost ancestral ones underscores this notion. Interestingly, we found that relocalized proteins show adaptations to the physico-chemical properties of their altered cellular environments through the selective fixation of amino acid substitutions.

A number of individual reports have revealed differences in subcellular localization of paralogous proteins in humans and other mammals (for example, [12]; see also references in [14]). Our study here motivates and warrants systematic surveys that address the role of subcellular adaptation in the functional diversification of mammalian (duplicate) genes. These should also aim to explore recent duplications (most duplications in the yeast genome - including those studied here - are old), in order to better understand the timing and selective pressures associated with this process. In fact, two individual recent cases from apes have shed initial light on the early stages of subcellular adaptation (L Rosso and colleagues, unpublished). These demonstrate that subcellular adaptation may indeed occur through both neolocalization (L Rosso and colleagues, unpublished) and sublocalization (L Rosso and colleagues, unpublished), and that subcellular adaptation may be accompanied or followed by adaptive changes of the biochemical function of the protein (L Rosso and colleagues, unpublished). Moreover, they show that subcellular shifts may be adaptive, driven by positive selection, and may occur through a few selected changes in specific (signal) sequences (consistent with our analysis of duplicated target sequences presented here), thus allowing for rapid retargeting of duplicate proteins during evolution.

We conclude that in addition to changes in their expression and biochemical function, selectively driven subcellular adaptation has played an important role for the functional diversification of duplicate genes and the emergence of new gene functions in both uni- and multicellular organisms. Thus, generally, investigating the subcellular phenotype of duplicate genes may provide valuable clues to their function and fate.

## Materials and methods

### *S. cerevisiae *WGD genes and other paralogs

We retrieved the gene IDs of 900 *S. cerevisiae *WGD paralogs (organized in 450 gene pairs) as well as the IDs, orthologs, and nucleotide/protein sequence of *K. waltii *orthologs from the supplemental data of [17]. Nucleotide and amino acid sequences for all *S. cerevisiae *WGD gene pairs and non-WGD paralogs (as defined by Ensembl gene family annotations) were retrieved from the Ensembl database, release 45 [35,36].

### Subcellular localization data

Subcellular localization data were retrieved from [37]. Only proteins unambiguously assigned to at least one of the 22 analyzed subcellular compartments were used. Another global protein localization data set [19,38] was used for comparison. Subcellular localizations (Figure 4) were predicted using Predotar [39,40].

### Non-synonymous substitution rates

We used MUSCLE 3.6 [41] to construct codon-based nucleotide alignments of *S. cerevisiae *WGD gene pairs and their corresponding *K. waltii *orthologous genes. To estimate the rate of non-synonymous changes, *d*_N_, along the different branches of the *S. cerevisiae*/*K. waltii *gene trees, we used the CODEML free-ratio model as implemented in the PAML 3.15 package [22].

### Phylogenetic reconstructions

We used a maximum likelihood approach, PROML, as implemented in the PHYLIP 3.67 software package, to reconstruct the phylogeny of the protein families (protein sequence alignment infiles were generated using MUSCLE 3.6).

### *S. cerevisiae *singletons

To identify proteins without paralogs (singletons), an all-against-all BLASTP similarity search (E-value = 0.1) was conducted. Proteins without hits against other proteins in this search were considered to be singletons.

### Gene ontology analysis

GO analyses were conducted using FatiGO [42,43].

### Gene expression analyses

*S. cerevisiae *gene expression levels - measured as the number of mRNA copies per cell - were retrieved from [33]. The average absolute protein abundance in pmol was retrieved from the literature (supplemental data of [44]). The fold difference of gene expression per gene pair was calculated as the absolute difference between the numbers of mRNA copies or protein concentration per gene normalized by the average mRNA copy number or protein concentration per gene pair.

### Isoelectric point and hydrophathy data

Hydrophathy and p*I *data were collected from the literature (supplemental data of [44]).

### Protein-protein interactions

Protein interactors for all proteins in D- and S-pairs were collected from the BioGRID repository [34,45].

## Abbreviations

ER, endoplasmic reticulum; GFP, green fluorescent protein; GO, Gene Ontology; SP, signal peptide; WGD, whole-genome duplication.

## Authors' contributions

ACM, NV and HK conceived and designed the experiments. ACM, NV and DB performed analysis. ACM and HK wrote the paper. All authors read and approved the final manuscript.

## Additional data files

The following additional data are available with the online version of this paper. Additional data file 1 is a table listing all whole genome duplicate pairs and relevant information for each member. Additional data file 2 contains the subcellular localizations of members from 45 *S. cerevisiae *protein families (each containing one WGD protein pair) and most parsimonious subcellular localization changes inferred based on the phylogeny.

## Supplementary Material

Additional data file 1 WGD proteins (in pairs), their subcellular localizations and other properties. Click here for file

Additional data file 2 The WGD pair is indicated in header for each tree. The compartments inferred to have been gained by neolocalization are underlined in red; those inferred to have resulted from sublocalization are underlined in green. Trees where subcellular relocalization events could not be inferred are labeled accordingly ('not clear'). Click here for file
