# Supplementary material for: Functional diversification of duplicate genes through subcellular adaptation of encoded proteins
Source: Genome Biol. 2008 Mar 12;9(3):R54. doi: 10.1186/gb-2008-9-3-r54 (PMC2397506; doi:10.1186/gb-2008-9-3-r54)

PMT2/PMT3 - No change

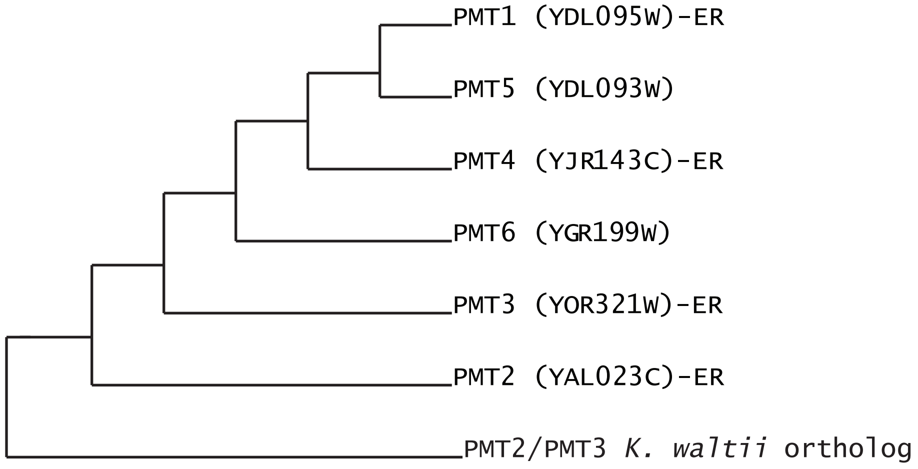

HHF1/HHF2 & HTB1/HTB2 - No change

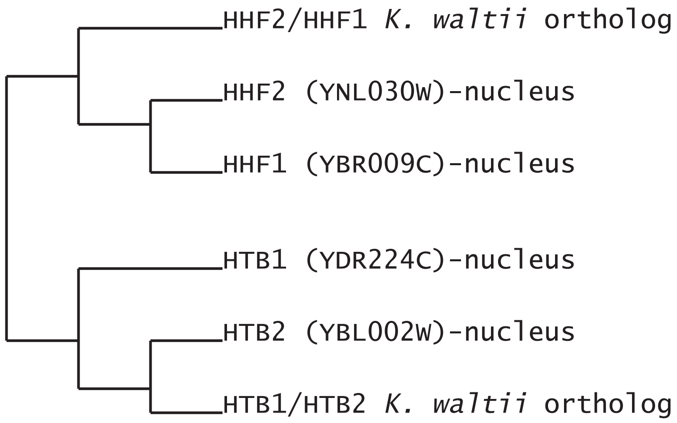

PRS4/PRS2 - No change

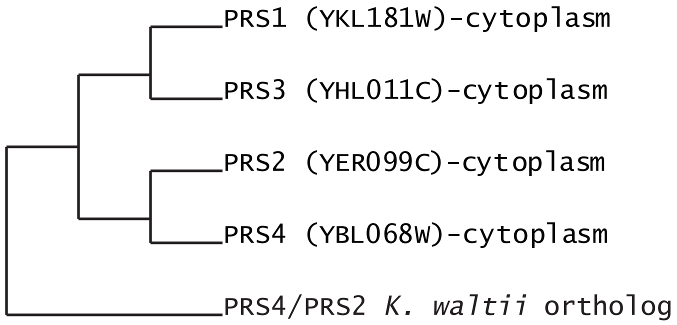

YBR014C/YDL010W & GRX1/TTR1 - Not clear

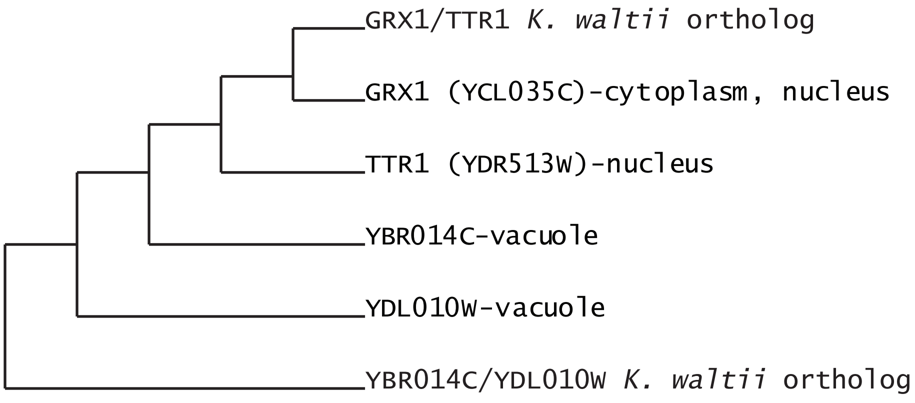

SSA3/SSA4 & SSB1/SSB2 - Neolocalization

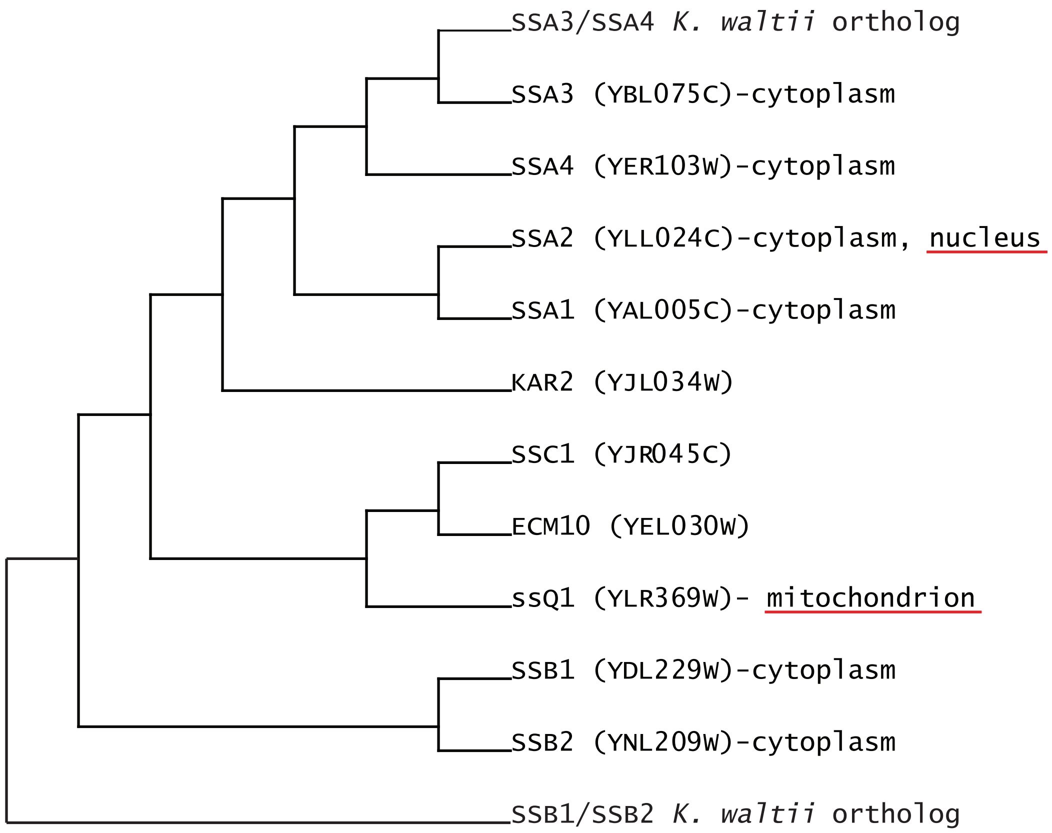

UBC4/UBC5 - Neolocalization + Sublocalization

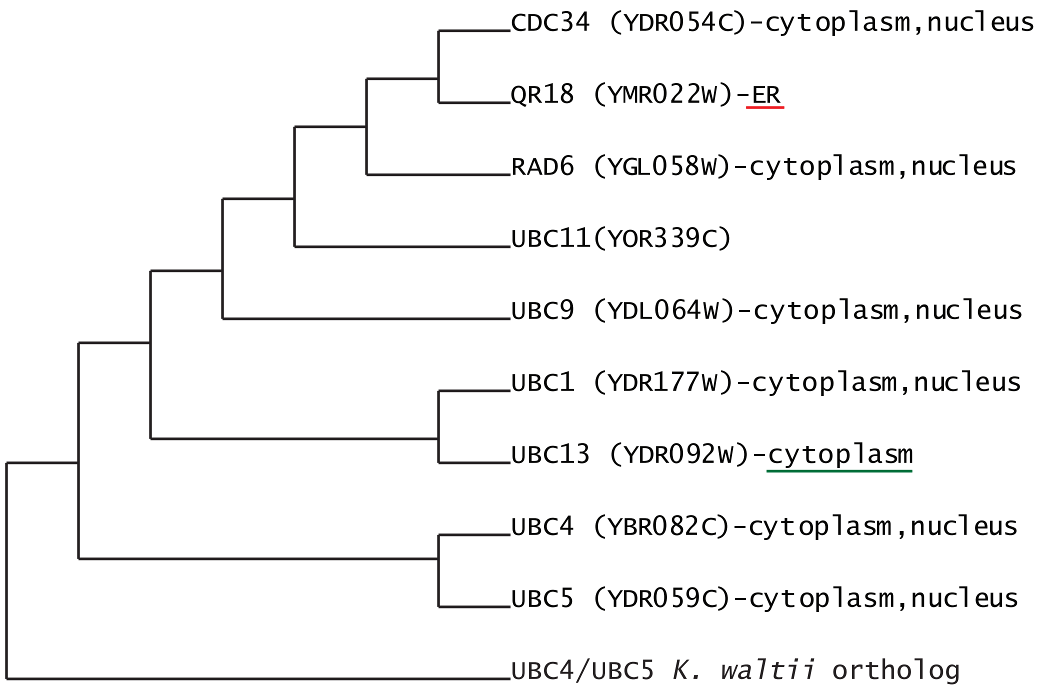

TPK2/TPK3 - Sublocalization

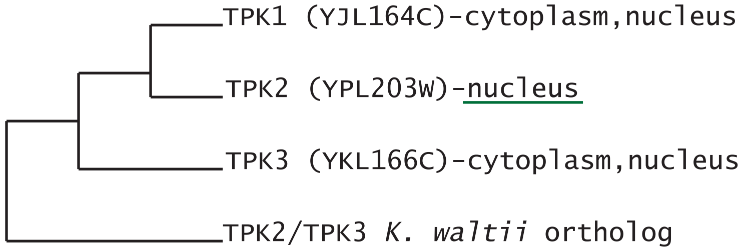

URA5/URA10 - Not clear

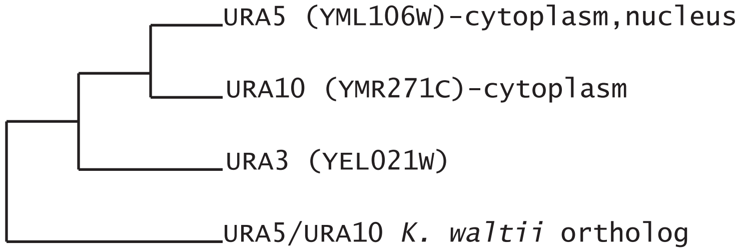

MSC2/ZCR1 - Not clear

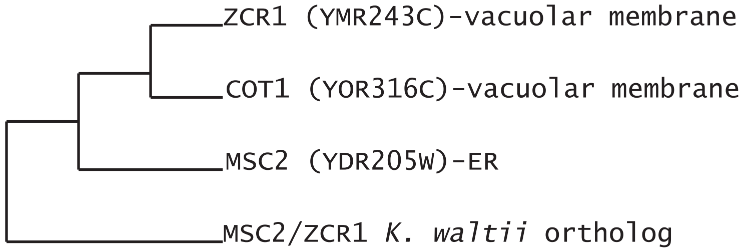

FAA1/FAA3 - Not clear

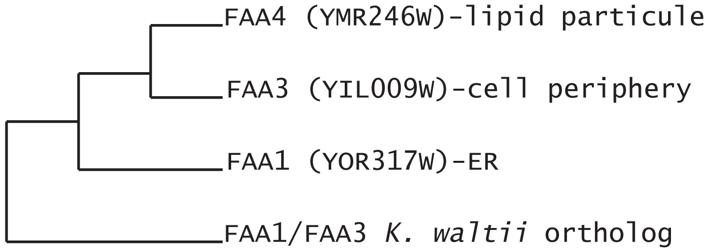

YML072C/YOR086C - No change

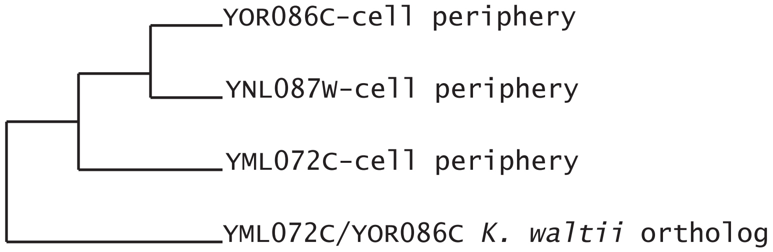

RPL24A/RPL24B - No change

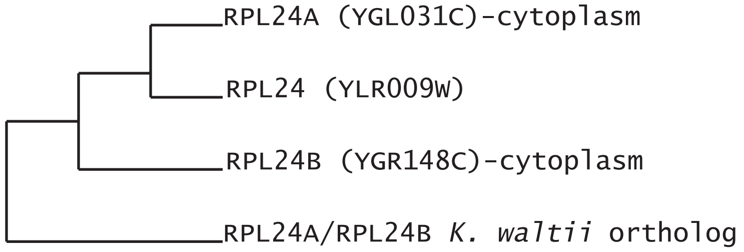

MEP2/MEP1 - No change

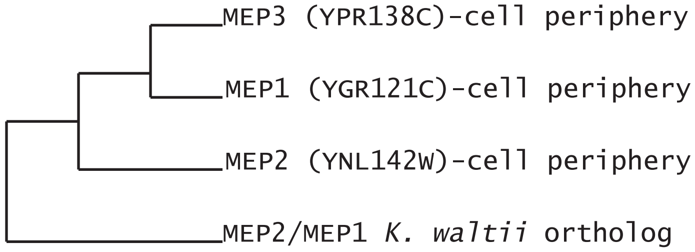

TDH1/TDH2 - No change

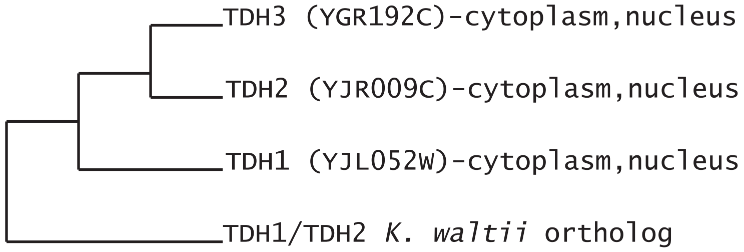

TRX3/TRX1 - Not clear

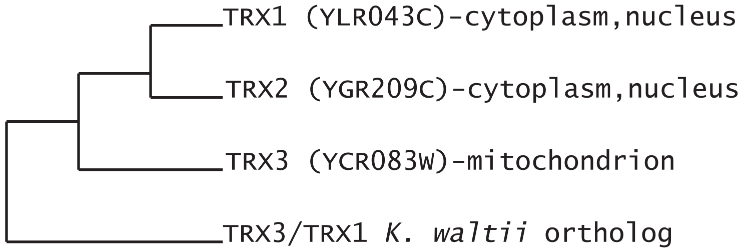

SFB3/SFB2 - No change

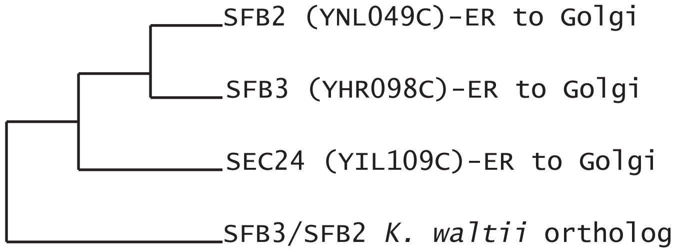

ENT1/ENT2 - No change

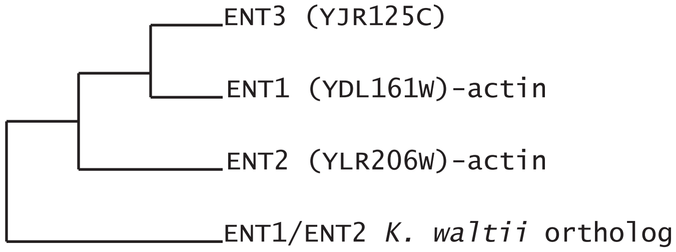

GIS2/AIR2 - Sublocalization

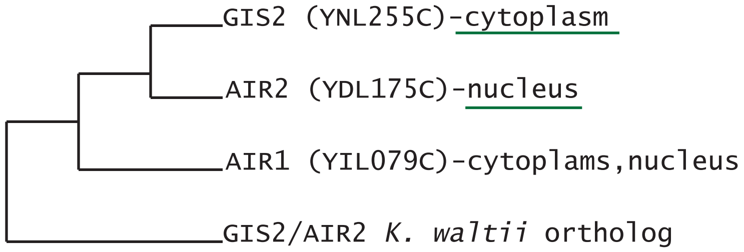

GRX5/GRX4 - Neolocalization

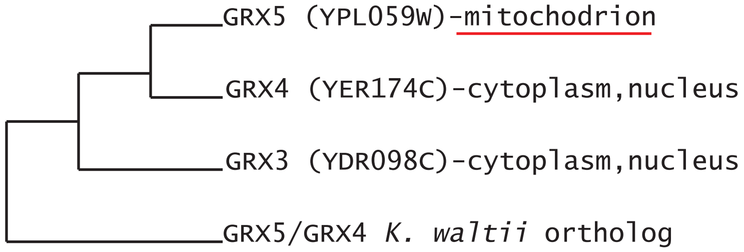

MSF1/YLR193C - No change

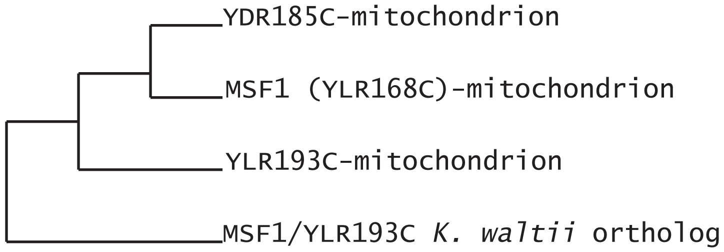

YNL274C/SER3 - Not clear

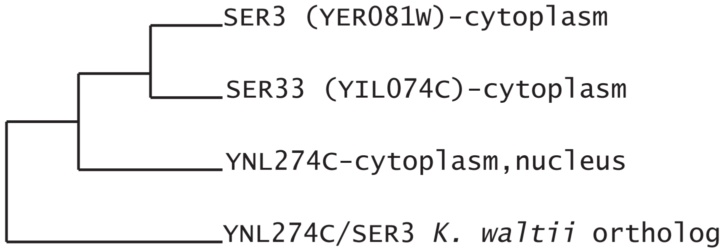

INP52/INP53 - Not clear

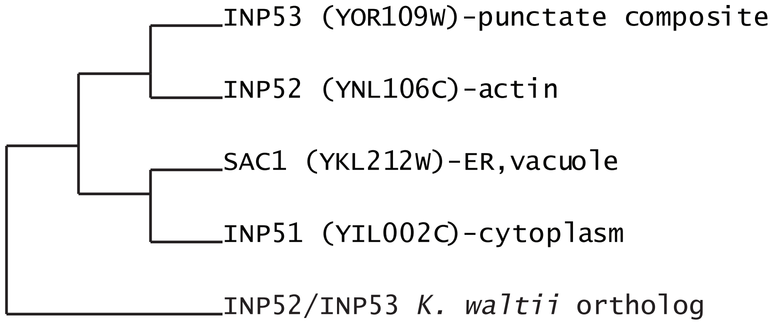

CIT1/CIT3 - Neolocalization

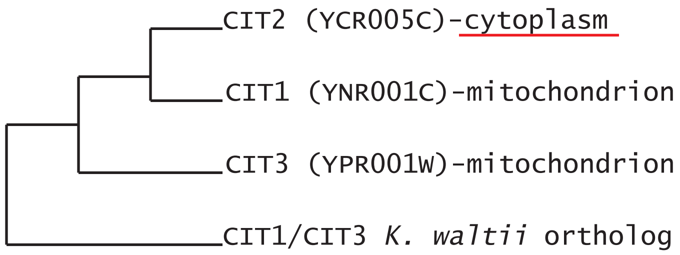

SUR4/FEN1 - No change

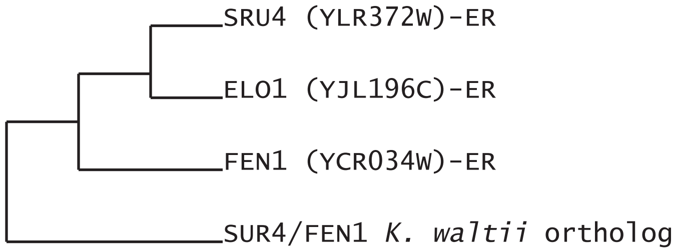

HST2/SIR2 - Not clear

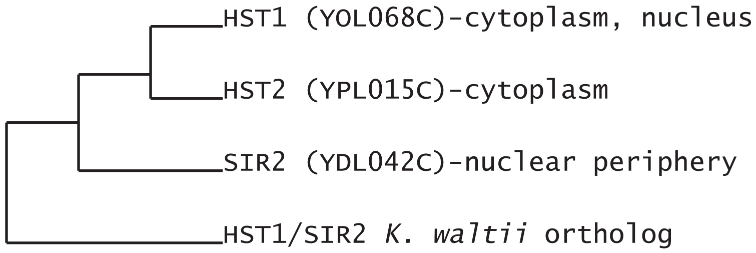

BDF2/BDF1 - Neolocalization

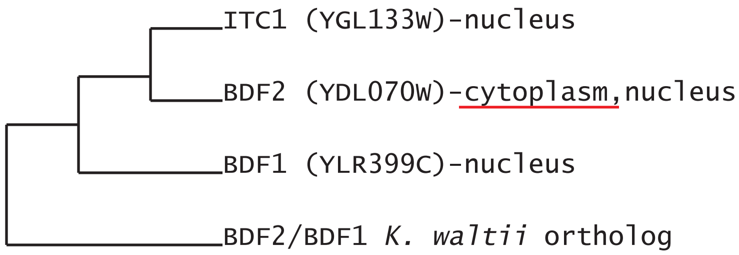

IMD3/IMD4 - No change

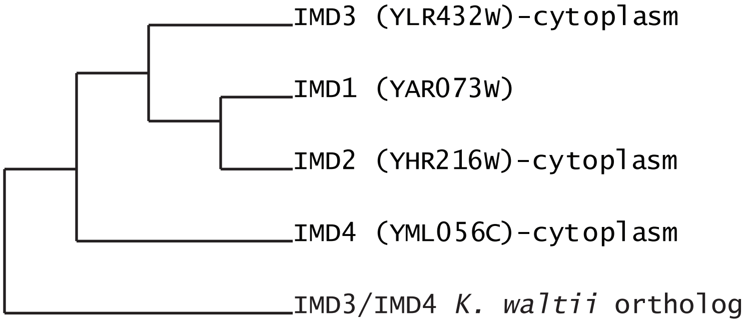

TUB1/TUB3 - Neolocalization + Sublocalization

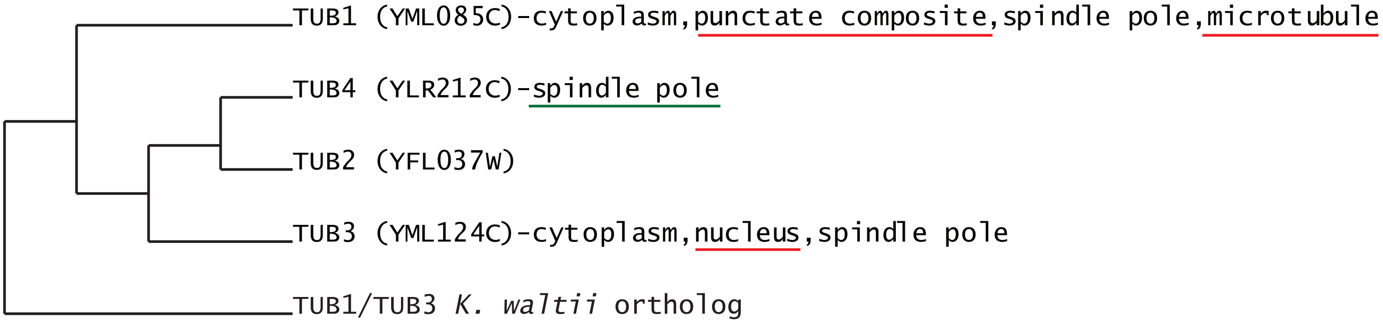

SSO2/SSO1 - Not clear

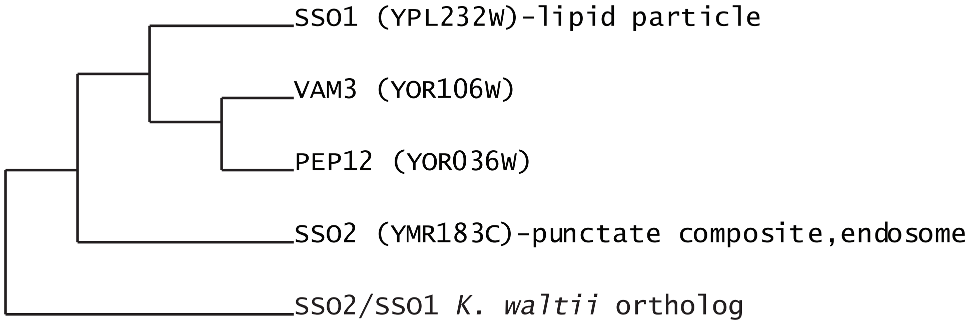

RAS2/RAS1 - Sublocalization

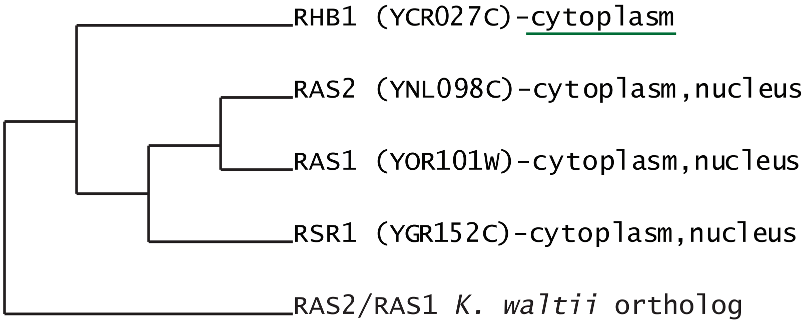

LSB1/PIN3 - Not clear

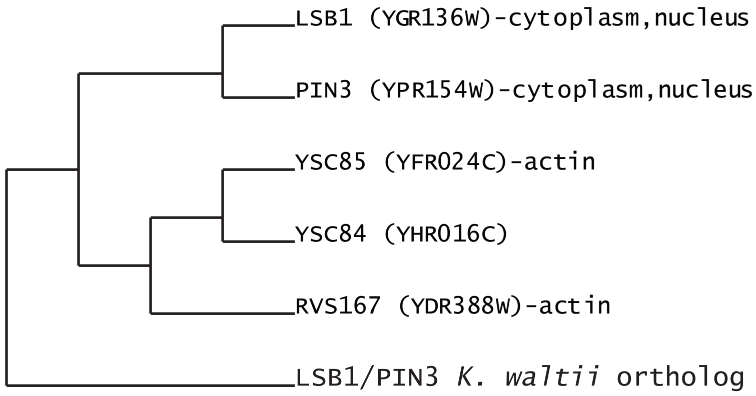

YCK1/YCK2 - Not clear

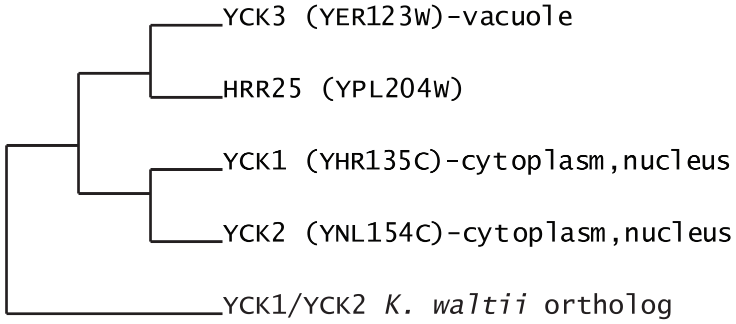

SDP1/MSG5 - Neolocalization

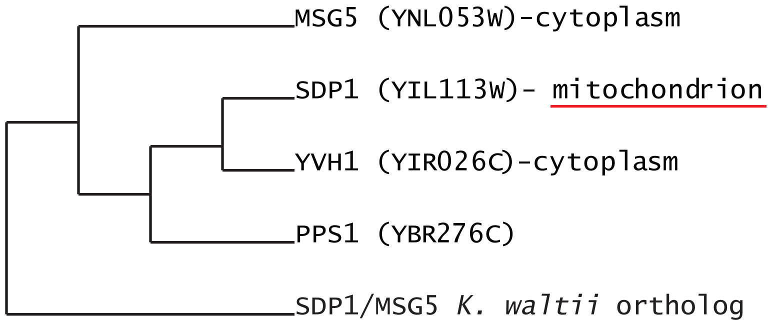

TIF1/TIF2 - Neolocalization

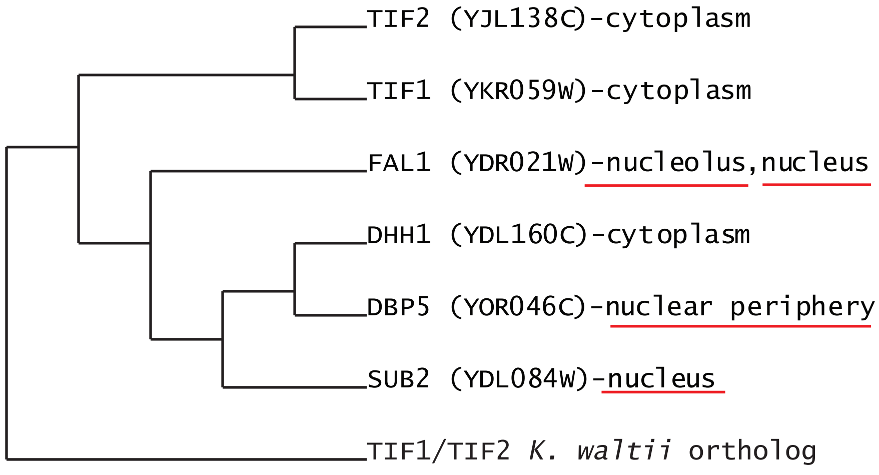

PPH21/PPH22 - Sublocalization + Neolocalization

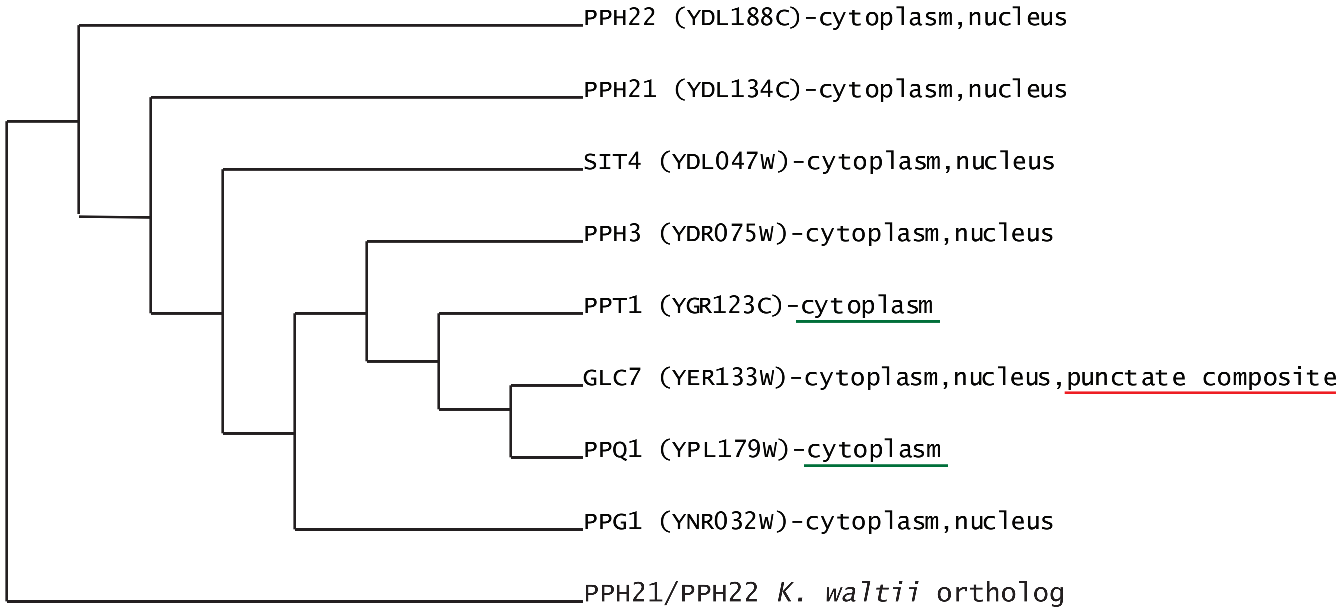

YPR1/GCY1 - No change

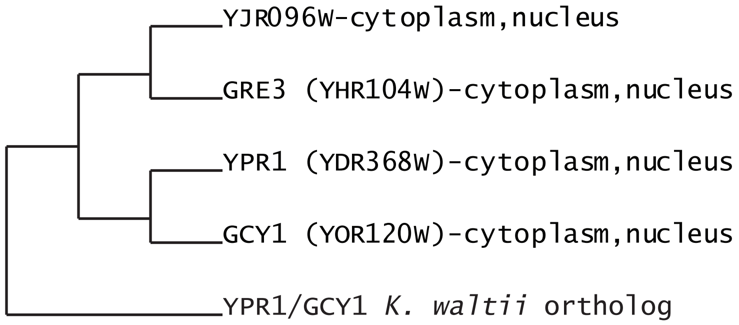

KRE2/KTR6 - No change

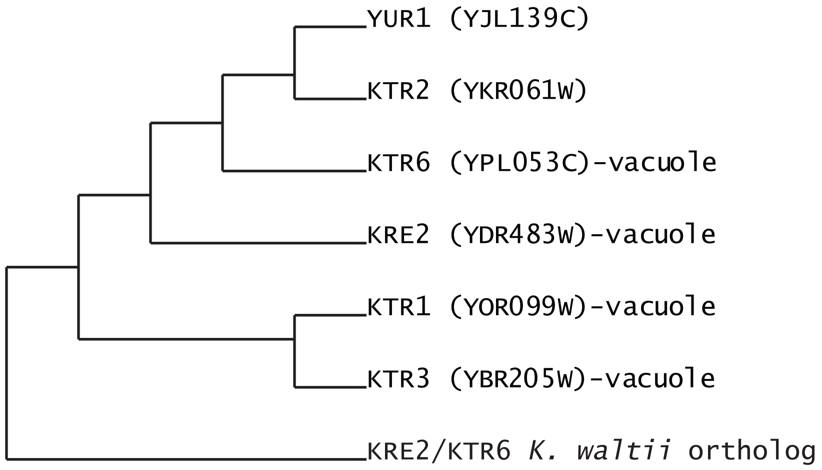

AGP1/GNP1 - Sublocalization + Neolocalization

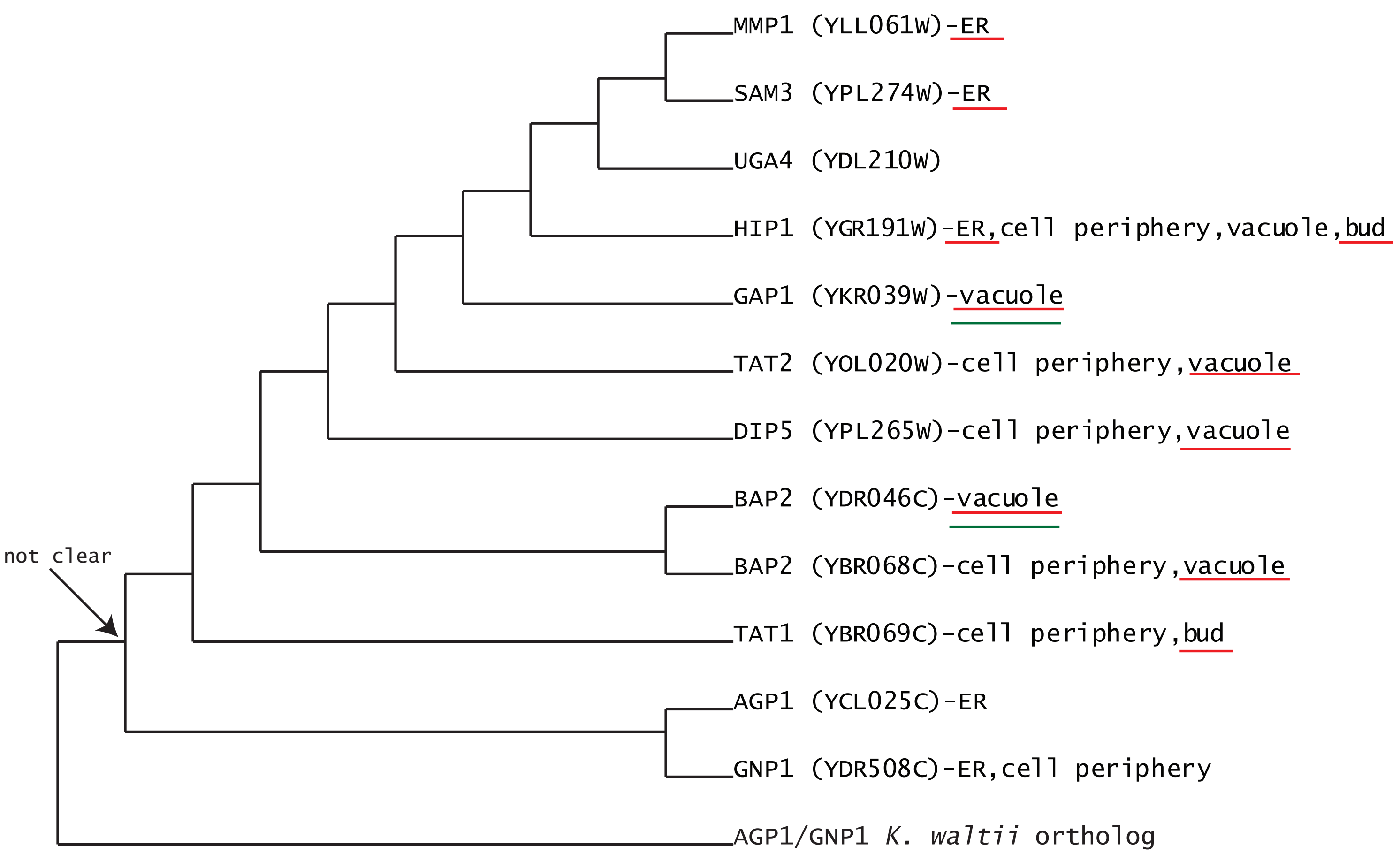

SOL4/SOL3 - Sublocalization

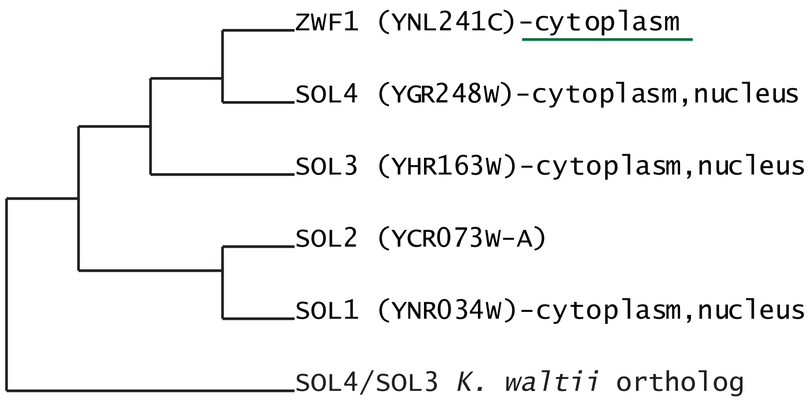

CLB3/CLB4 - Not clear

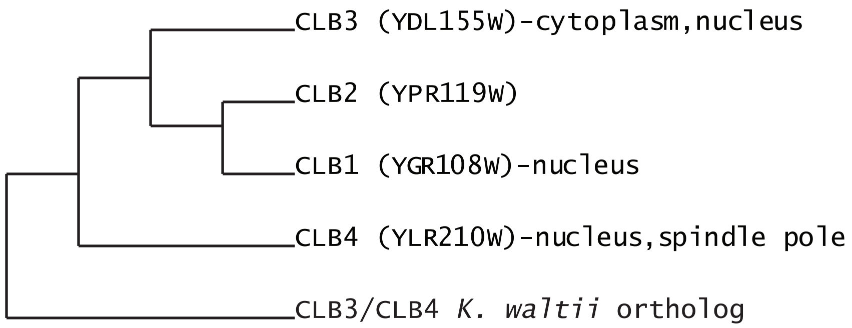

HTZ1/HTA1 - Neolocalization

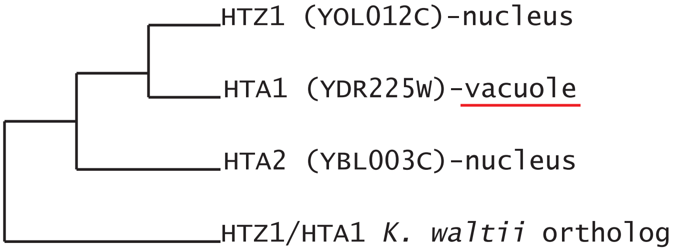

YIM1/AST1 - Not clear

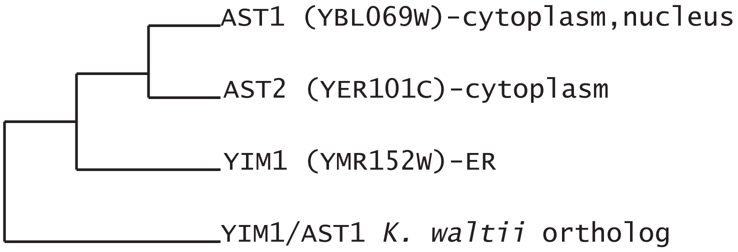

YCP4/PST2 - Neolocalization

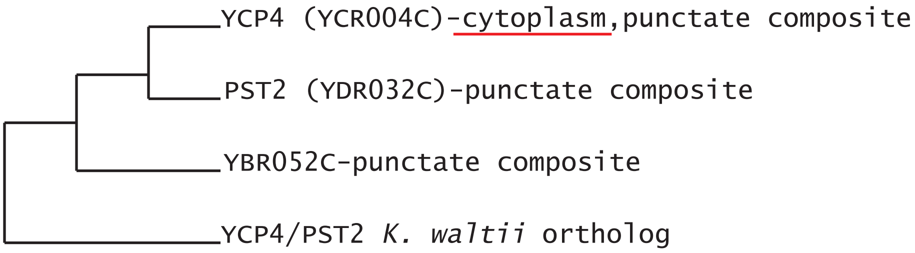

TEF1/SUP35 - No change

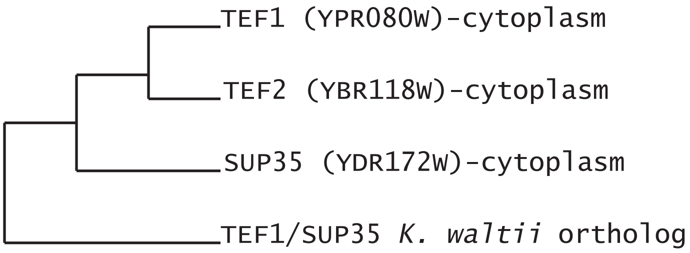

ENO1/ENO2 - No change

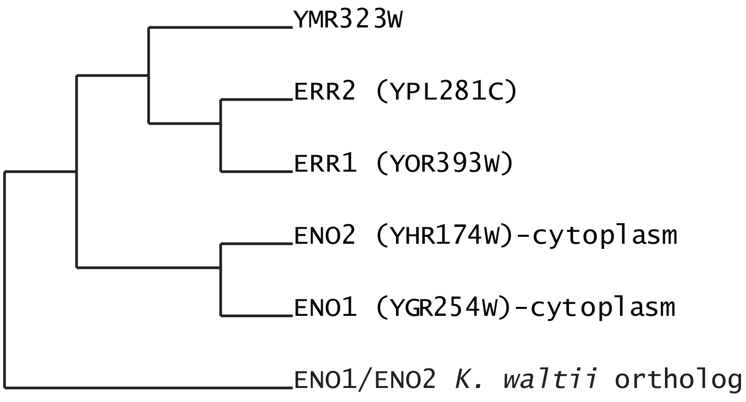

Supplement: Additional data file 2 — The WGD pair is indicated in header for each tree. The compartments inferred to have been gained by neolocalization are underlined in red; those inferred to have resulted from sublocalization are underlined in green. Trees where subcellular relocalization events could not be inferred are labeled accordingly ('not clear'). [file gb-2008-9-3-r54-S2.pdf]
